# Impact of locoregional treatment intensification in stampede high-risk M0 prostate cancer patients

**DOI:** 10.1016/j.brachy.2025.09.006

**Published:** 2025-10-14

**Authors:** Jackson Howell, Jonathan Tward

**Affiliations:** Huntsman Cancer Institute at the University of Utah, Salt Lake City, UT

**Keywords:** High-risk, Stampede, Prostate cancer, Brachytherapy

## Abstract

**INTRODUCTION:**

This study evaluates the efficacy of locoregional treatment intensification with combined modality therapy (CMT) for STAMPEDE high-risk (SHR) nonmetastatic (M0) prostate cancer. We compare metastasis-free survival (MFS) and overall survival (OS) between external beam radiation therapy (EBRT) with androgen deprivation therapy (ADT), CMT with a brachytherapy boost, and up-front surgery.

**MATERIAL AND METHODS:**

A retrospective cohort of 217 SHR patients from our institutional database was stratified by treatment modality: EBRT + ADT (*n* = 56), CMT (*n* = 61), and surgery (*n* = 100). Median ADT duration was 24 months for the EBRT + ADT group and 6 months for the CMT group. Primary outcomes were MFS and OS, analyzed using Cox proportional hazards regression and Fine-Gray competing risks models, adjusted for PSA, age, and Gleason group.

**RESULTS:**

CMT significantly improved adjusted MFS compared to EBRT + ADT (HR 0.41; *p* = 0.004). In contrast, up-front surgery did not significantly improve MFS over EBRT + ADT (HR 0.84; *p* = 0.471). Adjusted OS was also superior with CMT compared to EBRT + ADT (HR 1.98; *p* = 0.025). On subgroup analysis, the MFS benefit of CMT persisted for N0 patients but not for N1 patients. A key limitation is the retrospective, nonrandomized nature of the data.

**DISCUSSION AND CONCLUSIONS:**

Locoregional treatment intensification with CMT and de-intensified ADT offers significant oncologic benefits in men with SHR M0 prostate cancer, particularly in N0 patients. These findings support further investigation into combining brachytherapy and systemic therapy with de-intensified ADT in prospective trials.

## Introduction

Men with very high-risk localized or clinically node-positive prostate cancer have a high rate of progression and metastasis when treated with curative intent surgery or radiation therapy. Recently, investigators from the Systemic Therapy in Advancing or Metastatic Prostate Cancer: Evaluation of Drug Efficacy (STAMPEDE) trial performed a meta-analysis that identified a group of patients with nonmetastatic, hormone-sensitive prostate cancer who benefited from systemic therapy treatment intensification with the addition of abiraterone to androgen deprivation therapy (ADT) (1). These STAMPEDE-High Risk (SHR) patients were defined as having T_any_ N1 M0 disease or T_any_ N0 M0 with ≥2 of the following risk factors: initial prostate-specific antigen (iPSA) >40 ng/mL, Gleason score of 8–10, or T3-T4 disease. In 2025, the National Comprehensive Cancer Network (NCCN) adapted this definition for N0 patients as the new criteria for Very High-Risk localized prostate cancer. Approximately 85% of the patients evaluated in the STAMPEDE analyses also received prostate-directed radiation therapy. Therefore, the combination of ADT, androgen receptor signaling inhibitors (ARSIs), and prostate-directed radiation therapy has become a contemporary care standard.

Nevertheless, questions remain regarding the role of surgery and how to optimize radiation therapy, particularly in dose and volume intensification. The ideal duration of ADT was not a focus of the STAMPEDE investigators, and patients and oncologists remain interested in shortening the recommended duration of ADT whenever possible, due to its negative impact on quality of life.

Our study aimed to bridge these knowledge gaps by investigating the roles of surgery, pelvic lymph node radiation, and short-course ADT when using brachytherapy to boost in a retrospective outcomes analysis of our institution’s SHR population from the pre-STAMPEDE. There is reason to believe that further optimizing the radiation regimen, particularly with dose or volume intensification, could benefit patients in the post-STAMPEDE era.

## Material and methods

### Study design and patient population

This single-institution, retrospective cohort study utilized a prospectively maintained database of prostate cancer patients treated between 2005 and 2022 at our cancer center. Eligible patients were classified as SHR based on the following criteria: T_any_ N1 M0 disease or T_any_ N0 M0 with ≥ 2 risk factors: iPSA > 40 ng/mL, Gleason score of 8–10, or T3-T4 disease (1). Patients were stratified by nodal stage (N0 vs. N1) and definitive treatment modality.

### Treatment modalities

Patients received 1 of 3 definitive treatments: (1) External Beam Radiation Therapy (EBRT) with ADT (EBRT + ADT); (2) Combined Modality Therapy (CMT) utilizing EBRT, brachytherapy boost, and ADT; or (3) up-front surgery with radiation as indicated afterwards. It is our institutional practice to (1) utilize pelvic nodal radiation therapy (PNRT) when definitively radiating patients with high-risk and very high-risk prostate cancer and (2) to deintensify ADT duration when combining EBRT with a brachytherapy boost (2). Thus, patients treated with EBRT + ADT are commonly prescribed 24 months of ADT, while patients receiving CMT are prescribed 6 months.

### Data collection

Baseline demographic and treatment characteristics were recorded, including age at definitive treatment, iPSA, staging, Gleason Group (GG), ADT duration, and Charlson Comorbidity Index (CCI). Outcomes of interest were metastasis-free survival (MFS) and overall survival (OS).

### Statistical analysis

Kaplan-Meier survival analysis was performed for unadjusted MFS and OS comparisons. To account for potential confounding factors, a Fine-Gray competing risks regression was used to adjust MFS and OS for log_10_(iPSA), patient age, and GG. Cox proportional hazards regression was employed to identify risk factors for metastasis and death, stratified by definitive treatment modality and nodal status. Statistical analysis was conducted using STATA software (version 18), with statistical significance set at *p* < 0.05.

## Results

### Treatment modalities

A total of 217 patients met the study inclusion criteria. They were treated definitively using 3 primary modalities: EBRT + ADT (*n* = 56); CMT (*n* = 61); and up-front surgery with prostatectomy (*n* = 100). Table 1 presents the patients’ demographic and initial treatment characteristics, sorted by definitive treatment modality, and Table 2 specifies the radiation and androgen deprivation therapy regimens utilized.

Demographic differences were expected among the 3 treatment groups. Patients in the EBRT + ADT group were older and had higher median Charlson Comorbidity Indices (CCIs) than those in the CMT and surgery groups (*p* < 0.001), likely reflecting increased anesthesia risk. The average iPSA was highest in the CMT group, which had a preponderance of T3-T4 tumors not reaching statistical significance. The CMT and surgery groups demonstrated a preponderance of N0 patients that was not statistically significant.

The majority of our radiation patients received PNRT (82.1% in the EBRT + ADT group; 100% in the CMT group). Of the 117 patients who received definitive radiation, 3 (2.6%) were later treated with salvage radiation. Of the 100 patients definitively treated with surgery, 40 (40.0%) later received either postoperative or salvage radiation. The median ADT duration in the EBRT + ADT group was 24 months, whereas the median ADT duration in the CMT group was 6 months. Of the 40 patients in the surgery group who received postoperative or salvage RT, the median ADT duration was 6 months.

### Nodal status

Table 3 presents patient demographics and initial treatment information, organized by nodal status. Notable findings included higher T-stage, iPSA, and GG among N0 patients, consistent with the inclusion criteria of the SHR classification. Notably, ADT duration was evenly distributed between the nodal groups.

### Metastasis-free survival

**Fig. 1.**
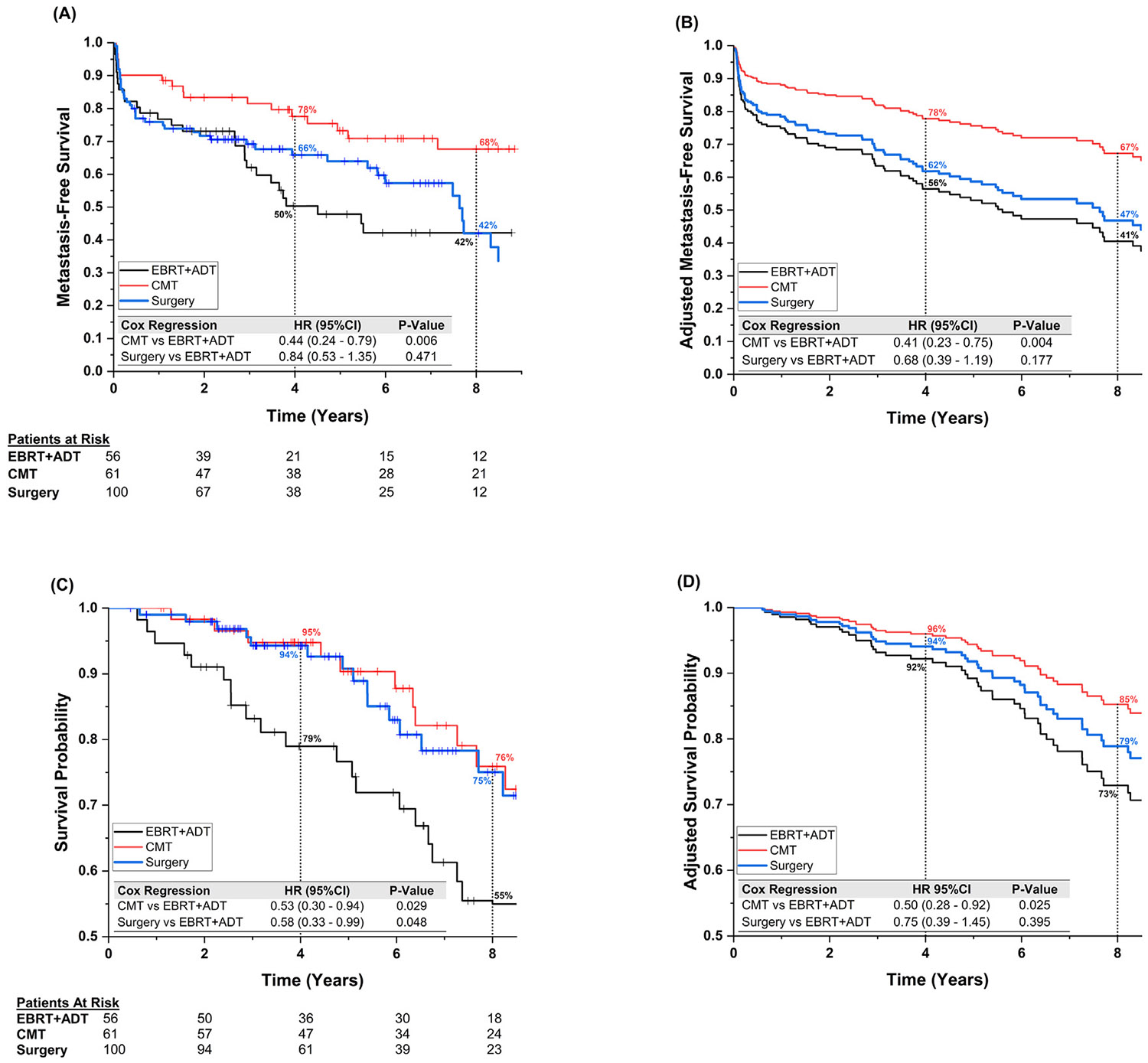
Comparison of unadjusted Kaplan-Meier curves (A and C) and adjusted cumulative incidence functions using Fine-Gray competing risks analysis (B and D) for metastasis-free survival (A and B) and overall survival (C and D) for STAMPEDE high-risk prostate cancer patients treated with various definitive modalities. Adjusted curves were controlled for patient age at definitive treatment, log10(iPSA), and Gleason Grade Group. ADT - Androgen Deprivation Therapy; CMT - Combined Modality Therapy (EBRT + Brachytherapy Boost + ADT), EBRT - External Beam Radiation Therapy; iPSA–Initial Prostate Specific Antigen; STAMPEDE - Systemic Therapy in Advancing or Metastatic Prostate Cancer: Evaluation of Drug Efficacy..

Figure 1A displays the unadjusted MFS in our patient cohort stratified by definitive treatment modality. Cox proportional hazard modeling showed a benefit of CMT over EBRT+ADT (HR 0.44, 95%CI [0.23–0.79], *p* = 0.006), but MFS did not significantly improve with surgery over EBRT+ADT (HR 0.84, 95%CI [0.52–1.35]; *p* = 0.471). MFS diverged early for the CMT group and sustained throughout follow-up compared to the surgery and EBRT+ADT groups (4-yr MFS = 77.6%, 65.9%, and 50.3%; 8-yr MFS = 67.6%, 42.0%, and 42.2%, respectively).

Figure 1B presents the adjusted MFS analysis after controlling for log_10_(iPSA), age, and GG. This demonstrated a consistent improvement in MFS for patients treated with CMT compared to EBRT+ADT (HR 0.41, 95%CI [0.23–0.75]; *P* = 0.004). However, the MFS improvement seen for surgery over EBRT+ADT was not statistically significant (HR 0.68, 95%CI [0.39–1.19], *p* = 0.177).

### Overall survival

Figure 1C displays the unadjusted OS of our patient cohort stratified by definitive treatment modality. Both the surgery (HR 0.58, 95%CI [0.33–0.99], *p* = 0.048) and CMT (HR 0.53, 95%CI [0.30–0.94], *p* = 0.029) groups demonstrated significant improvements in OS compared to the EBRT +ADT group. OS was closely approximated between the surgery and CMT groups at 4 (94.3% and 94.8%, respectively) and 8 years (75.5% and 75.0%, respectively) compared with the EBRT+ADT group (79.0% and 55.5%, respectively).

Figure 1D shows the adjusted OS analysis for our treatment groups, controlling for log_10_(iPSA), age, and GG. Only CMT demonstrated a consistent benefit over EBRT + ADT (HR 1.98, 95%CI [1.09–3.60], *p* = 0.025).

### Metastasis risk

Table 4 presents the calculated risks of metastasis and death associated with clinical variables of interest, stratified by nodal status. For N0 patients, using CMT for definitive treatment was found to reduce the metastatic risk compared to EBRT+ADT (HR 0.35, 95%CI [0.14–0.85]; *p* = 0.021). This benefit persisted in the combined cohort (HR 0.41, 95%CI [0.23–0.75]; *p* = 0.004) but was not statistically significant in the N1 population (HR 0.90, 95%CI [0.38–2.12]; *p* = 0.806). Up-front surgery demonstrated no MFS benefit comparable to EBRT+ADT. The presence of a GG5 tumor was also found to increase the risk of metastatic spread relative to GG4 tumors in the N0 population (HR 1.95, 95%CI [1.02–3.75], *p* = 0.044) and the total cohort (HR 1.99, 95%CI [1.22–3.24], *p* = 0.006). Age at the last biopsy and log_10_(iPSA) were not significantly correlated with metastasis risk.

### Death risk

Contrary to the findings for metastatic risk, a statistically significant improvement in death risk was only observed for CMT over EBRT + ADT in the total cohort (HR 1.98, 95%CI [1.09–3.60], *p* = 0.025). This effect was not observed in the subgroup analysis of nodal stage. However, log_10_(iPSA) (HR 1.34, 95%CI [1.03–1.73], *p* = 0.030) and age at the last positive prostate biopsy (HR 1.07, 95%CI [1.03–1.11], *p* = 0.001) correlated with a higher risk of death in N0 patients. As with metastasis risk, the presence of GG5 tumors appeared to increase death risk in the N0 (HR = 2.42, 95%CI [1.29–4.55], *p* = 0.006) and total cohorts (HR = 2.27, 95%CI [1.34–3.84], *p* = 0.002) compared to the presence of GG4 primary disease.

## Discussion

Our investigation sought to retrospectively analyze the outcomes of various first-line treatment modalities in patients with SHR prostate cancer from the pre-STAMPEDE era to determine if there was potential for further optimization of local therapy. Our institutional experience with this patient population is distinctive. We have consistently used PNRT in our NCCN high-risk patients and a short-course ADT for brachytherapy patients for some time, giving us extended follow-up for this unique patient cohort from the pre-STAMPEDE era. We have demonstrated that locoregional treatment intensification with a brachytherapy boost is superior to EBRT alone in adjusted and unadjusted MFS and OS, despite de-intensification of ADT to 6 months in these patients. However, the MFS benefit persisted only in the N0 subgroup. Up-front surgery demonstrated an unadjusted OS advantage over EBRT + ADT; however, this advantage disappeared when adjusting for critical prognostic factors.

The STAMPEDE trial brought a paradigm shift to the treatment of metastatic prostate cancer, where systemic therapy alone had been the standard of care, by demonstrating a significant benefit in failure-free and overall survival with prostate-directed therapy in patients with low metastatic burden metastatic prostate cancer (3-5). These findings were subsequently supported by the STOPCAP meta-analysis and were speculated to be driven by immune modulation and hindrance of the premetastatic niche (5,6). The SHR classification was a direct result of this trial and provides unique treatment considerations for oncologists. These patients are at an exceptional risk of local, nodal (>44% risk of microscopic nodal disease by the Briganti Nomogram), and distant recurrence (7-9). Thus, optimizing treatment for this population requires integrating treatment principles from both the local and metastatic paradigms (10).

The superiority of CMT with de-intensified ADT over EBRT with standard ADT in our analysis is a substantial finding. It builds upon work by Patel et al. (11) comparing oncological outcomes between NCCN high- and very high-risk prostate cancer patients treated with EBRT and ≥ 24 months of ADT versus CMT and ≥12 months of ADT. In this pooled, multi-institutional analysis, there was no statistically significant difference in unadjusted prostate cancer-specific survival (HR 1.08, 95%CI (0.31–9.88), *P* = 0.522) or freedom from distant metastasis (HR 0.58, 95%CI (0.14–2.37), *P* = 0.449) between the 2 arms for SHR patients. This is strong evidence that ADT can be de-intensified without significant worsening of oncologic outcomes. Kishan et al. (12) published similar results in the NCCN high-risk prostate cancer population, where definitive radiation modalities demonstrated improved prostate cancer-specific survival and lower rates of distant metastasis—the greatest benefit being in the brachytherapy group—compared to surgery.

Additionally, the MFS of our CMT group was similar to that of the control group from the STAMPEDE analysis by Attard et al., who received EBRT alongside 3 years of ADT (6-year MFS = 69%) (1). It is unclear exactly how adding standard-of-care abiraterone to our CMT regimen would change oncologic outcomes. However, these results suggest that evaluating CMT with de-escalated ADT duration in combination with abiraterone may be a potential target for prospective clinical trials to challenge the standard of care.

ADT results in long-term reductions in quality of life (QOL) related to fatigue, sexual function, and physical/mental aptitude (13,14). For many men, reducing ADT duration is highly desirable, particularly if oncologic outcomes are not impacted. Kishan and colleagues recently published a multi-institutional analysis suggesting that optimal ADT duration may be 12 months when combined with a brachytherapy boost in NCCN high-risk patients (15). Additionally, the recently published results of the Tri-Modality Therapy with I-125 Brachytherapy, External Beam Radiation Therapy, and Short- or Long-Term Hormone Therapy for High-Risk Localized Prostate Cancer (TRIP) trial from Japan suggested equivalence between long-course (30 months) and short-course (6 months) ADT when combined with LDR brachytherapy boost in high-risk prostate cancer (16).

In the pre-STAMPEDE era, guidance on ADT duration and EBRT regimens for the treatment of cN1 prostate cancer was limited, particularly in the setting of brachytherapy boosts. NCCN guidelines of that era gave wide latitude. Many institutions extrapolated high-risk and very high-risk data treatment outcomes to patients with cN1 disease. Concurrently, multiple studies suggested that ADT could be de-escalated in the setting of brachytherapy boosts, as summarized in the contemporary American Brachytherapy guidelines on the topic (17). Among them, a retrospective national database analysis of that era called into question the benefit of ADT of any duration in men with high-risk prostate cancer (18). Because of the uncertainty in the data, our institution took what was at the time a middle ground, offering these patients 6 months of ADT when treatment utilized a brachytherapy boost.

However, the addition of a brachytherapy boost has consequences. In an analysis of patient-reported QOL data from NCCN high-and very high-risk prostate cancer patients treated at our institution, adding a brachytherapy boost significantly worsened urinary irritative and obstructive toxicity symptoms at 6 months compared to men who received EBRT + ADT (2). This acute worsening resolved by 1 year after treatment completion. The clinical implication of this QOL analysis, in combination with the data presented here, is that appropriately selected men in this exceptionally high-risk category may now have the option of de-escalating ADT duration in exchange for short-term worsening of urinary symptoms without compromising oncologic outcomes.

There are, however, multiple limitations to our analysis. First, the benefits of CMT appeared to be driven by the N0 subgroup (66% of the cohort). Adding a brachytherapy boost demonstrated no metastasis or death risk benefits in the N1 population. This could be secondary to underpowering, as there were only 17 and 23 N1 patients in the CMT and EBRT+ADT groups, respectively.

Additionally, as is common in retrospective studies, statistically significant differences in baseline characteristics were observed among the 3 treatment groups. The surgical and CMT groups generally exhibited more favorable age and comorbidity characteristics, likely due to anesthesia risk considerations associated with the invasive procedure involved. These initial disparities were anticipated to manifest as survival differences in the unadjusted OS analysis, which was the sole instance where upfront surgery showed a statistically significant survival benefit over EBRT + ADT. Consequently, we presented more statistically robust adjusted MFS and OS data, and it revealed no significant difference between surgery and EBRT + ADT.

The majority of our radiation patients underwent PNRT, a practice not universally adopted in our field and specifically not advised for the control group in Attard’s analysis of SHR patients (1). PNRT has been a contentious issue among radiation oncologists for decades (19). The underlying rationale for PNRT is that a portion of cN0 patients may have microscopic disease in the pelvic nodes, and treating these nodes could enhance oncological outcomes, ideally with minimal added side effects in the IMRT era. Randomized clinical studies have not consistently demonstrated PNRT’s benefits, thus it is not considered standard practice. The RTOG 9413 trial sought to elucidate PNRT’s role in high-risk prostate cancer patients. Initially, this study showed improved 4-year progression-free survival (PFS) with PNRT combined with neoadjuvant ADT (20); however, 10-year data indicated this approach was less effective (10-yr PFS 28% vs. 30%) and more toxic (late grade 3+ GI toxicities: 7% vs. 2%) compared to prostate-only radiation with adjuvant ADT (21). Given these uncertainties, our institution has favored treating nodal volumes in patients at high risk of nodal disease. The upcoming release of RTOG 0924 data is expected to provide more definitive guidance on this matter.

## Conclusions

This single-institution, retrospective analysis of prospectively gathered data in men with SHR prostate cancer from the pre-STAMPEDE era demonstrated improved adjusted and unadjusted MFS and OS for men treated with CMT utilizing only 6 months of ADT compared with men receiving EBRT along with 24 months of ADT. Given these findings, we support investigating EBRT with a brachytherapy boost and a de-intensified ADT duration (12 months or less) and ARSIs as an alternative to the CHAARTED-derived treatment targets and ADT durations for eligible men with SHR prostate cancer (11,12,15).

## Figures and Tables

**Table 1:** Patient demographics and initial treatment characteristics sorted by definitive treatment modality.

	EBRT + ADT *N* = *56 (25.8%)*	CMT *N* = *61 (28.1%)*	Surgery *N* = *100 (46.1%)*	Total *N* = *217 (100.0%)*	Test
Patient demographics					
*Age [Median (IQR)]*	70.7 (64.2–76.4)	66.7 (61.7–72.2)	63.3 (56.9–68.0)	65.2 (59.1–71.3)	*p* < 0.001*
*CCI [Median (IQR)]*	1.0 (0.0–2.0)	0.0 (0.0–1.0)	0.0 (0.0–0.0)	0.0 (0.0–1.0)	*p* < 0.001*
*Ethnicity [N (%)]*					*p* = 0.171
*Asian*	1 (1.8%)	0 (0.0%)	1 (1.0%)	2 (0.9%)	
*Black*	0 (0.0%)	1 (1.6%)	0 (0.0%)	1 (0.5%)	
*Hawaiian*	2 (3.6%)	0 (0.0%)	0 (0.0%)	2 (0.9%)	
*Native American*	0 (0.0%)	0 (0.0%)	1 (1.0%)	1 (0.5%)	
*Other*	1 (1.8%)	2 (3.3%)	1 (1.0%)	4 (1.8%)	
*Unknown*	9 (16.1%)	5 (8.2%)	6 (6.0%)	20 (9.2%)	
*White*	43 (76.8%)	53 (86.9%)	91 (91.0%)	187 (86.2%)	
Initial treatment characteristics					
*iPSA in ng/mL [Median (IQR)]*	21.9 (10.2–68.4)	24.3 (9.9–48.0)	21.3 (8.0–55.8)	21.9 (9.2–57.0)	*p* = 0.011*
T-Stage [N (%)]					*p* = 0.138
*T1-T2*	28 (50.0%)	21 (34.4%)	49 (49.0%)	98 (45.2%)	
*T3-T4*	28 (50.0%)	40 (65.6%)	51 (51.0%)	119 (54.8%)	
*N-Stage [N (%)]*					*p* = 0.314
*N0*	33 (58.9%)	44 (72.1%)	67 (67.0%)	144 (66.4%)	
*N1*	23 (41.1%)	17 (27.9%)	33 (33.0%)	73 (33.6%)	
*Gleason grade grouping [N (%)]*					*P* = 0.910
*1–3*	9 (16.1%)	8 (13.1%)	14 (14.0%)	31 (14.3%)	
*4*	19 (33.9%)	25 (41.0%)	41 (41.0%)	85 (39.2%)	
*5*	28 (50.0%)	28 (45.9%)	45 (45.0%)	101 (46.5%)	
*ADT duration in months [Median (IQR)]*	24.0 (18.0–28.0)	6.0 (6.0–6.0)	n/a	6.0 (6.0–24.0)	*p* < 0.001*

* *P* < 0.05; ADT = androgen deprivation therapy; CCI = Charlson comorbidity index; CMT = combined modality therapy (EBRT + brachytherapy boost + ADT); EBRT = external beam radiation therapy; iPSA = initial prostate specific antigen; IQR = interquartile range.

**Table 2:** Radiation and androgen deprivation therapy regimens.

	EBRT + ADT *N* = *56*	CMT *N* = *61*	Surgery^a^ *N* = *100*
Prostate/fossa dose and fractionation			
*No prostate or fossa RT*	0 (0%)	0 (0%)	60 (60.0%)
*64.8–70.2 Gy (1.8–2.0 Gy/Fx)*	1 (1.7%)	0 (0%)	34 (34.0%)
*72.0–75.6 Gy (1.8–2.0 Gy/Fx)*	11 (19.6%)	0 (0%)	0 (0%)
*77.0–81.0 Gy (1.8–2.0 Gy/Fx)*	29 (51.8%)	0 (0%)	1 (1.0%)
*70 Gy (2.5 Gy/Fx)*	10 (17.9%)	0 (0%)	0 (0%)
*36.25 Gy (7.25 Gy/Fx)*	2 (3.6%)	0 (0%)	0 (0%)
*Pelvic nodal dose* + *brachytherapy boost*	0 (0%)	61 (100.0%)	0 (0%)
*Unknown dose*	3 (5.4%)	0 (0%)	5 (5.0%)
Pelvic nodal dose and fractionation			
*No pelvic nodal RT*	7 (12.5%)	0 (0%)	61 (61.0%)
*< 45 Gy at 1.8–2.0 Gy/ Fx*	0 (0%)	1 (1.6%)	0 (0%)
*45–50.4 Gy at 1.8–2.0 Gy/ Fx*	43 (76.7%)	60 (98.4%)	33 (33.0%)
*> 50.4–59.4 Gy at 1.8–2.0 Gy/ Fx*	3 (5.4%)	0 (0%)	1 (1.0%)
*Unknown dose*	3 (5.4%)	0 (0%)	5 (5%)
*Median length of ADT received*	24 months	6 months	6 months
*Peri-RT ADT regimens*			
*No ADT or No RT*	0 (0%)	0 (0%)	62 (62.0%)
*Unknown*	19 (33.9%)	11 (18.0%)	7 (7.0%)
*3–6 months*	6 (10.7%)	39 (64.0%)	21 (21.0%)
*7–12 months*	2 (3.6%)	1 (1.6%)	4 (4.0%)
*13–24 months*	17 (30.4%)	7 (11.5%)	4 (4.0%)
*> 24 months*	12 (21.4%)	3 (4.9%)	2 (2.0%)

a All RT courses were adjuvant/salvage. ADT = androgen deprivation therapy; CMT = combined modality therapy (EBRT + brachytherapy boost + ADT); EBRT = external beam radiation therapy; RT = radiation therapy; Fx = fraction.

**Table 3:** Patient demographics and initial treatment characteristics sorted by nodal status.

	N0 *N* = *144 (66.4%)*	N1 *N* = *73 (33.6%)*	Total *N* = *217 (100.0%)*	Test
Patient demographics				
*Age [Median (IQR)]*	66.0 (61.0–72.6)	64.4 (57.7–69.8)	65.2 (59.1–71.3)	*p* = 0.032
*CCI [Median (IQR)]*	0.0 (0.0–1.0)	0.0 (0.0–1.0)	0.0 (0.0–1.0)	*p* = 0.398
*Ethnicity (%)*				*p* = 0.792
*Asian*	1 (0.7%)	1 (1.4%)	2 (0.9%)	
*Black*	1 (0.7%)	0 (0.0%)	1 (0.5%)	
*Hawaiian*	2 (1.4%)	0 (0.0%)	2 (0.9%)	
*Native American*	1 (0.7%)	0 (0.0%)	1 (0.5%)	
*Other*	2 (1.4%)	2 (2.7%)	4 (1.8%)	
*Unknown*	12 (8.3%)	8 (11.0%)	20 (9.2%)	
*White*	125 (86.8%)	62 (84.9%)	187 (86.2%)	
Initial Treatment Characteristics				
*iPSA in ng/mL [Median (IQR)]*	40.4 (11.5–69.2)	13.9 (8.1–27.1)	21.9 (9.2–57.0)	*p* = 0.004^a^
*T-Stage [N (%)]*				*p* < 0.001^a^
*T1-T2*	49 (34.0%)	49 (67.1%)	98 (45.2%)	
*T3-T4*	95 (66.0%)	24 (32.9%)	119 (54.8%)	
*Definitive Treatment Modality [N (%)]*				*p* = 0.314
*EBRT* + *ADT*	33 (22.9%)	23 (31.5%)	56 (25.8%)	
*CMT*	44 (30.6%)	17 (23.3%)	61 (28.1%)	
*Surgery*	67 (46.5%)	33 (45.2%)	100 (46.1%)	
*Gleason Grade Grouping [N (%)]*				*p* < 0.001^a^
*1–3*	10 (6.9%)	21 (28.8%)	31 (14.3%)	
*4*	65 (45.1%)	20 (27.4%)	85 (39.2%)	
*5*	69 (47.9%)	32 (43.8%)	101 (46.5%)	
*ADT Duration in Months [Median (IQR)]*	6.0 (6.0–24.0)	6.0 (6.0–24.0)	6.0 (6.0–24.0)	*p* = 0.561

a *p* < 0.05; ADT = androgen deprivation therapy; CCI = charlson comorbidity index, CMT = combined modality therapy (EBRT + brachytherapy boost + ADT); EBRT = external beam radiation therapy, iPSA = initial prostate specific antigen, IQR = interquartile range.

**Table 4:** Death and metastasis risk factor analysis of STAMPEDE high-risk prostate cancer patients.

	Death risk									Metastasis risk								
	N0			N1			Total			N0			N1			Total		
	*N* = *144 (66.4%)*			*N* = *73 (33.6%)*			*N* = *217 (100.0%)*			*N* = *144 (66.4%)*			*N* = *73 (33.6%)*			*N* = *217 (100.0%)*		
	*HR*	*95% CI*	*p-Value*	*HR*	*95% CI*	*p-Value*	*HR*	*95% CI*	*p-Value*	*HR*	*95% CI*	*p-Value*	*HR*	*95% CI*	*p-Value*	*HR*	*95% CI*	*p-Value*
**Definitive treatment modality**																		
*EBRT* + *ADT (reference)*	–	–	–	–	–	–	–	–	–	–	–	–	–	–	–	–	–	–
*CMT*	0.65	[0.32 – 1.33]	0.239	0.47	[0.11 – 2.07]	0.319	0.50	[0.28 – 0.92]	0.025^a^	0.35	[0.14 – 0.85]	0.021^a^	0.9	[0.38 – 2.12]	0.806	0.41	[0.23 – 0.75]	0.004^a^
*Surgery*	1.35	[0.59 – 3.10]	0.473	0.48	[0.13 – 1.77]	0.274	0.75	[0.39 – 1.45]	0.395	0.73	0.33 – 1.62]	0.445	1.08	[0.49 – 2.38]	0.853	0.68	[0.39 – 1.19]	0.177
**log10(iPSA)**	1.34	[1.03 – 1.73]	0.030^a^	0.55	[0.29 – 1.06]	0.072	1.05	[0.85 – 1.28]	0.668	1.27	[0.98 – 1.65]	0.068	0.97	[0.61 – 1.55]	0.897	1.09	[0.89 – 1.33]	0.404
**Age at Last Positive Biopsy Gleason grade group**	1.07	[1.03 – 1.11]	0.001^a^	0.98	[0.93 – 1.04]	0.559	1.04	[1.00 – 1.07]	0.024^a^	0.98	[0.94 – 1.01]	0.216	1.01	[0.96 – 1.05]	0.777	0.98	[0.95 – 1.00]	0.081
*2*	0.65	[0.08 – 5.05]	0.678	0.28	[0.05 – 1.76]	0.177	0.86	[0.25 – 3.00]	0.815	0.79	[0.08 – 7.56]	0.839	0.18	[0.02 – 1.47]	0.109	0.46	[0.10 – 2.08]	0.311
*3*	0.68	[0.09 – 5.31]	0.714	n/a	n/a	n/a	0.28	[0.04 – 2.12]	0.219	1.38	[0.26 – 7.35]	0.707	1.12	[0.43 – 2.93]	0.815	1.76	[0.80 – 3.86]	0.158
*4 (Reference)*	–	–	–	–	–	–	–	–	–	–	–	–	–	–	–	–	–	–
*5*	2.42	[1.29 – 4.55]	0. 006^a^	1.48	[0.50 – 4.40]	0.482	2.27	[1.34 – 3.84]	0.002^a^	1.95	[1.02 – 3.75]	0.044^a^	1.52	[0.71 – 3.28]	0.282	1.99	[1.22 – 3.24]	0.006^a^

a *P* < 0.05; ADT = androgen deprivation therapy; CMT = combined modality therapy (EBRT + Brachytherapy Boost + ADT), EBRT = external beam radiation therapy, iPSA = initial prostate specific antigen.

## Data Availability

Research data supporting this publication are available from the Researchers upon request.
